# Olfactory Hallucinations as the Initial Symptom of Anti-Leucine-Rich Glioma-Inactivated 1 Antibody Encephalitis: A Case Report and Review of the Literature

**DOI:** 10.7759/cureus.113841

**Published:** 2026-08-03

**Authors:** Huiyao Xiang, Moushan Cai

**Affiliations:** 1 Department of Neurology, The First College of Clinical Medical Science, China Three Gorges University, Yichang, CHN; 2 Department of Neurology, China Three Gorges University, Yichang, CHN

**Keywords:** anti-lgi-1 antibodies, autoimmune encephalitis, facial-arm dystonic episodes, immunotherapy, olfactory hallucinations, psychiatric symptoms

## Abstract

Anti-leucine-rich glioma-inactivated 1 (LGI-1) antibody encephalitis is an autoimmune encephalitis mediated by LGI-1 antibodies that target proteins on the surface of neuronal cell membranes. Its clinical manifestations are diverse, but olfactory hallucinations rarely present as the initial symptom. We report the case of a 61-year-old man who initially presented with olfactory hallucinations and abdominal discomfort lasting six months. This was rapidly followed by memory impairment, twitching at the right corner of the mouth, and upper limb spasms. A follow-up brain MRI scan revealed abnormal signals in the bilateral hippocampi, and both serum and cerebrospinal fluid (CSF) tests were positive for LGI-1 antibodies, leading to a diagnosis of anti-LGI-1 antibody-associated encephalitis. Following high-dose steroid pulse therapy, the patient achieved complete remission of symptoms. This report suggests that olfactory hallucinations may be an early manifestation of the disease involving the parahippocampal gyrus and the uncus. In middle-aged men presenting with new-onset olfactory hallucinations or similar symptoms, antibody screening should be performed as early as possible.

## Introduction

Anti-leucine-rich glioma-inactivated 1 (LGI-1) encephalitis is an important subtype of autoimmune encephalitis that has garnered significant attention in recent years because of its distinctive clinical manifestations and reversible pathophysiology. This disease most commonly affects middle-aged and elderly men and is the second most common type of autoimmune encephalitis after anti-N-methyl-D-aspartate receptor (NMDAR) encephalitis. Although approximately 64.4% of cases occur in men, the disease can affect women as well [1]. The core clinical syndrome includes short-term memory impairment, psychiatric and behavioral abnormalities, seizures, and characteristic faciobrachial dystonic seizures (FBDS), the latter of which are considered a highly specific hallmark of the disease [2,3]. Importantly, these symptoms typically present in distinct temporal phases rather than developing gradually with age. Prodromal manifestations, such as mood disturbances or olfactory hallucinations, may appear weeks to months before the full-blown syndrome develops, including cognitive impairment and the emergence of FBDS [4]. Hallucinations are not uncommon in this disease, but they are predominantly auditory or visual. Cases in which olfactory hallucinations serve as the initial or primary symptom are extremely rare [5].

The parahippocampal gyrus and the uncus are the sites of the primary olfactory cortex, where the LGI-1 protein is highly expressed [6]. Anti-LGI-1 antibodies are pathogenic autoantibodies that serve as both diagnostic biomarkers and direct mediators of synaptic dysfunction by disrupting the LGI-1-ADAM22/23 trans-synaptic complex, rather than merely passive markers of disease [7]. Olfactory hallucinations are not merely "epileptic auras" but may indicate an early, selective attack by antibodies on the limbic system [6]. This report describes the clinical course of a patient with anti-LGI-1 encephalitis presenting with olfactory hallucinations as the initial symptom. We discuss key diagnostic and therapeutic considerations, informed by the literature, to enhance clinicians' awareness of this rare initial presentation. Early immunotherapy, primarily with high- dose corticosteroids, remains the cornerstone of treatment and is associated with favorable, often reversible outcomes [8].

## Case presentation

A 61-year-old man who presented with episodic olfactory hallucinations and abdominal discomfort for six months was admitted on October 13, 2024. He reported recurrent olfactory abnormalities during this period without any apparent trigger. He described the odors as unpleasant smells, such as a "burnt smell" or "gasoline smell." Each episode lasted from a few seconds to several minutes and occurred more than 10 times daily. The episodes were often accompanied by abdominal discomfort without nausea or vomiting, and the symptoms resolved spontaneously after each episode. There was no history of fever, headache, or diarrhea before the onset of symptoms, nor had he experienced episodes of altered consciousness or limb convulsions.

The patient had previously sought care at a local hospital, but no definitive diagnosis had been established, and no specific treatment had been provided. He had a history of lumbar disc herniation surgery; otherwise, his medical history was unremarkable. General physical and neurological examinations revealed no abnormalities. Laboratory results indicated a sodium level of 135.4 mmol/L (normal range: 137-147 mmol/L) and a chloride level of 98.8 mmol/L (normal range: 99-110 mmol/L) among the electrolyte measurements. All other tests, including a complete blood count, serum potassium, a muscle enzyme panel, liver and kidney function tests, and thyroid function tests, were within normal limits (Table 1).

**Table 1:** Blood-related tests and results

Parameters	Results	Unit	Reference range
Complete blood count			
White blood count	6.78	10^9^/L	3.5-9.5
Lymphocytes	42.9	%	20-50
Neutrophils	51.5	%	40-75
Hemoglobin	141	g/L	130-175
Platelets	138	10^9^/L	125-350
Serum electrolytes			
Potassium	4.89	mmol/L	3.5-5.5
Sodium	135.4	mmol/L	137-147
Chloride	98.8	mmol/L	99-110
Muscle enzyme profile test			
Creatine kinase	58	U/L	50-310
Lactate dehydrogenase	211	U/L	120-250
Renal function test			
Creatinine	79	umol/L	57-97
Urea	6.7	mmol/L	3.1-8.0
Uric acid	312	umol/L	208-428
Liver function test			
Aspartate aminotransferase	35.5	U/L	15-40
Alanine aminotransferase	24.5	U/L	9-50
Total protein	77	g/L	65-85
Albumin	48	g/L	40-55
Total bilirubin	5.5	umol/L	0-26.0
Direct bilirubin	2.6	umol/L	0-6.8
Thyroid function test			
Triiodothyronine	1.10	nmol/L	0.89-2.45
Thyroxine	79.92	nmol/L	62.68-150.84
Thyroid-stimulating hormone	3.23	uIU/ml	0.35-4.94

**Figure 1:**
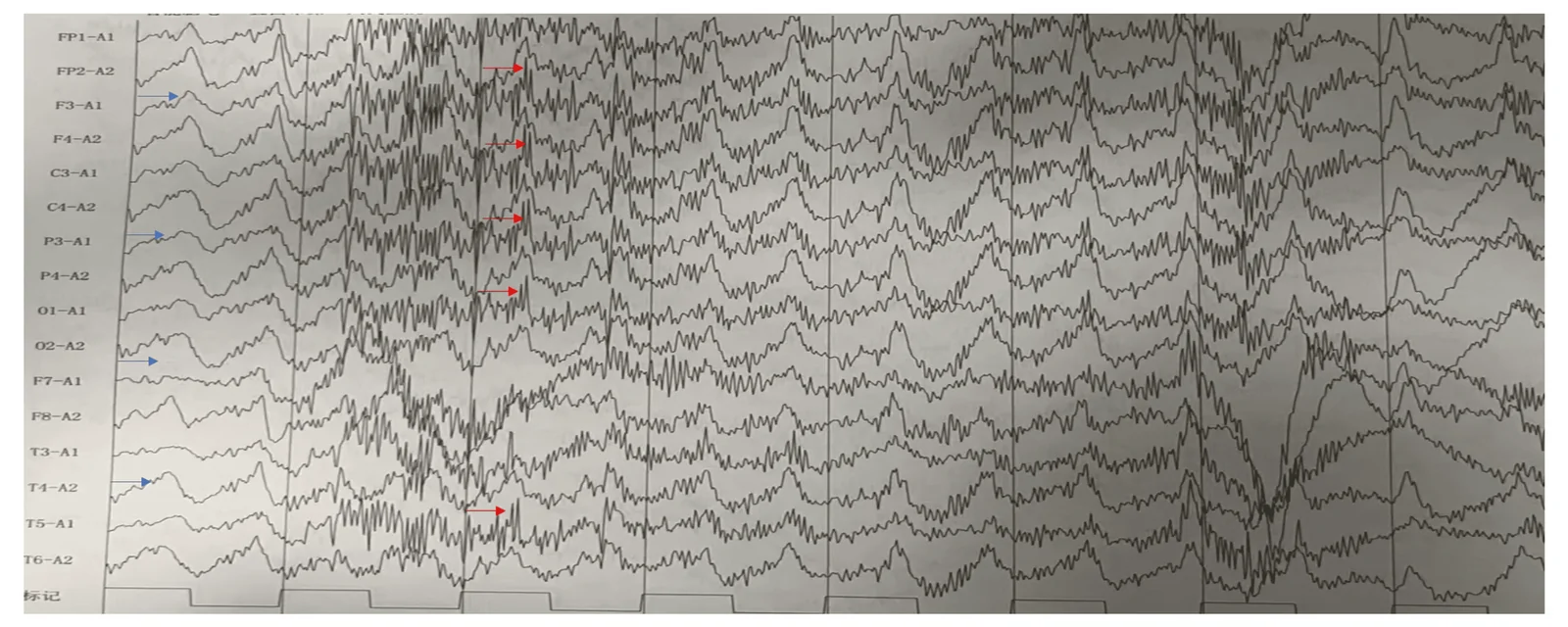
Video EEG findings The image shows diffuse slow-wave (blue arrow) activity accompanied by medium-to-high-amplitude sharp-spike waves (red arrow). Diffuse slow-wave activity indicates generalized cerebral dysfunction, while the sharp-spike waves represent epileptiform discharges suggestive of cortical irritability EEG: electroencephalogram

Scores on the Hamilton Anxiety and Depression Scale [9] were 14 on the anxiety subscale and 13 on the depression subscale, indicating mild anxiety and depression. Imaging studies revealed no significant abnormalities on abdominal CT. Plain and enhanced brain MRI, cerebral vascular MRA, and MRV showed no abnormalities. Video electroencephalography (EEG) (Figure 1) showed diffuse slow-wave activity accompanied by medium-to-high-amplitude sharp waves. The combination of episodic olfactory hallucinations, abdominal discomfort, and epileptiform discharges on EEG led to our initial diagnostic impression of symptomatic epilepsy involving the mesial temporal structures. Consequently, we made a provisional clinical diagnosis of symptomatic epilepsy and treated the patient with sodium valproate, together with anti-anxiety and antidepressant therapy. Following treatment, the olfactory hallucinations subsided, and the frequency of seizures decreased to one or two times per day. The patient reported symptomatic improvement and declined further testing, including screening for autoimmune encephalitis antibodies. He was discharged on October 30, 2024.

The day after discharge (November 1, 2024), the family noticed that the patient had developed memory impairment, manifested as intermittent failure to recognize family members, forgetting recent events, and repetitive questioning, accompanied by personality changes, including reduced speech, emotional detachment, and loss of interest, as well as sleep disturbances. More troubling were the physical symptoms: the patient developed involuntary twitching of the right corner of the mouth and right upper limb, which manifested as dystonic postures. Each episode lasted a few seconds and occurred 10 to 20 times daily, without any loss of consciousness. As these symptoms progressively worsened, the patient was readmitted on January 1, 2025. Neurological examination revealed that the patient was alert and had clear speech. However, recent memory was significantly impaired, while remote memory remained intact. The patient's ability to perform calculations was also impaired. For example, he was unable to complete serial subtraction of 7 from 100 (100 - 7 = 93, 93 - 7 = ?). No other abnormalities were detected during the neurological examination.

Laboratory tests showed normal levels of tumor markers, an autoantibody panel, a five-item immunological panel, human immunodeficiency virus antigen and antibody, syphilis antibodies, and hepatitis B surface antigen. Cerebrospinal fluid (CSF) analysis showed no abnormalities in opening pressure, cell count, protein, glucose, or chloride levels. Additionally, tests for acid-fast staining, India ink staining, Cryptococcus capsular antigen, and microbial cultures were all negative. Testing for central nervous system demyelinating antibodies and paraneoplastic syndrome-associated antibodies in both CSF and serum yielded normal results, and CSF next-generation sequencing (NGS) showed no evidence of infection, thereby excluding infectious etiologies. However, anti-LGI-1 antibody testing in the serum and CSF using a cell-based assay (CBA) was positive, with titers of 1:320 and 1:10, respectively (Table 2).

**Table 2:** Blood and CSF-related tests and results CSF: cerebrospinal fluid

Parameters	Results	Unit	Reference range
Blood			
Carbohydrate antigen 50	＜1.00	U/ml	＜21.96
Alpha-fetoprotein	3.11	ng/ml	＜7.42
Carcinoembryonic antigen	1.70	ng/ml	＜4.1
Carbohydrate antigen 125	22.68	U/ml	＜37.22
Carbohydrate antigen 153	11.71	U/ml	＜27.96
Carbohydrate antigen 199	＜2.30	U/ml	＜27.50
Carbohydrate antigen 242	＜0.10	U/ml	＜10.00
Neuron-specific enolase	6.25ng/ml	ng/ml	＜16.01
Autoantibodies 19 items	Negative		Negative
Immune function panel 5 items	Negative		Negative
Antibodies to human immunodeficiency virus antigens	0.04 S/CO		0-0.99
Syphilis antibodies	0.01 S/CO		0-0.99
Hepatitis B surface antigen	0.01	IU/mL	0-0.05
Central nervous system demyelinating antibodies (anti-myelin oligodendrocyte glycoprotein antibodies, anti-autoantibodies to aquaporin 4 antibodies, anti-glial fibrillary acidic protein antibodies, anti-myelin basic protein antibodies)	Negative		Negative
Oligoclonal bands	Negative		Negative
Autoimmune encephalitis antibodies 33 items	LGI-1 1:320		Negative
Paraneoplastic syndrome-related antibodies (anti⁃neuronal nuclear antibody type⁃1, anti⁃neuronal nuclear antibody type⁃2, collapsin response⁃mediator protein⁃5, anti-neuronal nuclear antibodies-3, purkinje cell cytoplasmic antibody type 2 antibodies)	Negative		Negative
CSF			
Leukocyte count	3	10^6^/L	0-8
Protein	26.50	mg/dl	15-45
Glucose	2.30	mmol/L	2.2-3.9
Chloride	126.7	mmol/L	118-132
Antacid staining	Negative		Negative
Ink staining	Negative		Negative
Cryptococcal capsular antigen	Negative		Negative
Cultures	Negative		Negative
Next-generation sequencing	Negative		Negative
Central nervous system demyelinating antibodies (anti-myelin oligodendrocyte glycoprotein antibodies, anti-autoantibodies to aquaporin 4 antibodies, anti-glial fibrillary acidic protein antibodies, anti-myelin basic protein antibodies)	Negative		Negative
Oligoclonal bands	Negative		Negative
Autoimmune encephalitis antibodies 33 items	LGI-1 1:10		Negative
Paraneoplastic syndrome-related antibodies (anti⁃neuronal nuclear antibody type⁃1, anti⁃neuronal nuclear antibody type⁃2, collapsin response⁃mediator protein⁃5, anti-neuronal nuclear antibodies-3, purkinje cell cytoplasmic antibody type 2 antibodies)	Negative		Negative

**Figure 2:**
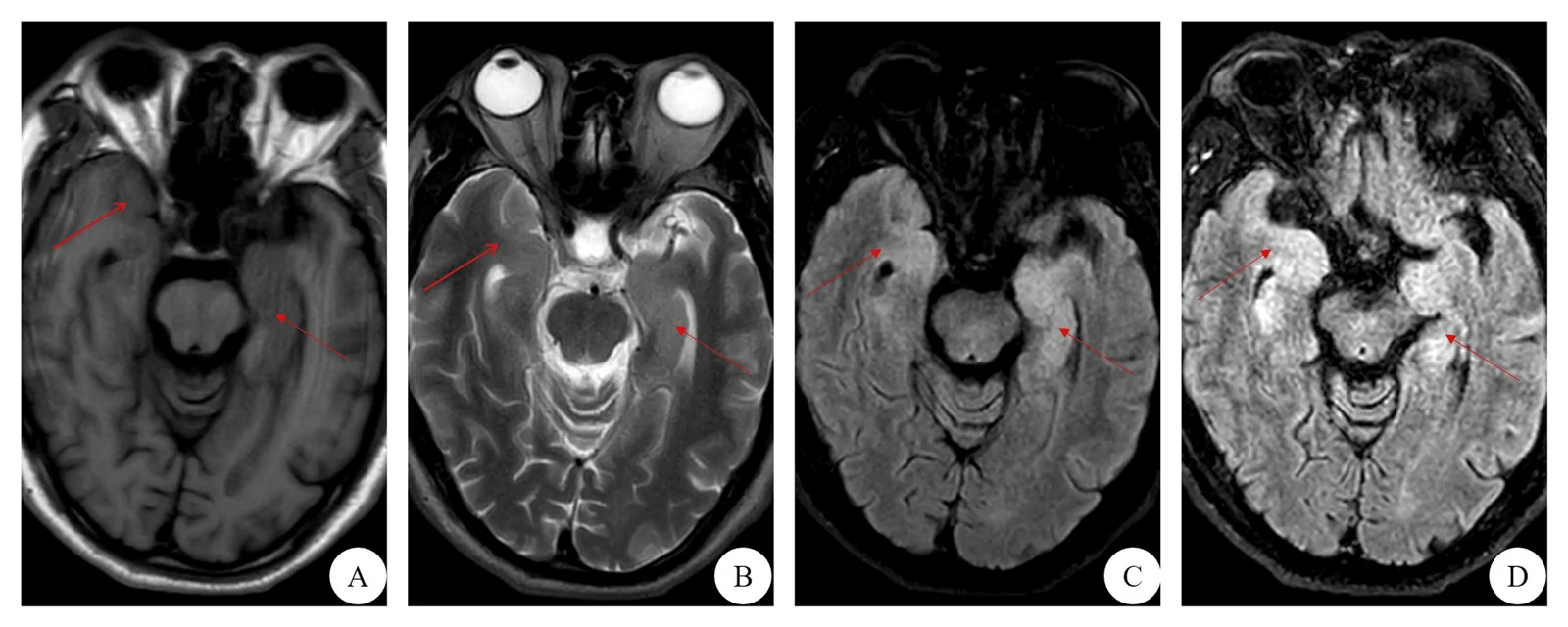
Brain MRI findings (A) In the T1-weighted image, the bilateral hippocampal lesions show slightly hypointense signals (the red arrow). (B) In the T2-weighted image, the lesions appear as hyperintense signals (the red arrow). (C) In the FLAIR image, the lesions also present as hyperintense signals (the red arrow). (D) In the enhanced scan image, there is no significant enhancement of the lesion (the red arrow). Bilateral hippocampal T2/FLAIR hyperintensity indicates limbic inflammation, which is a characteristic but not universal finding in anti-LGI-1 encephalitis. The absence of contrast enhancement suggests a non-neoplastic, inflammatory etiology rather than a tumor MRI: magnetic resonance imaging; FLAIR: fluid-attenuated inversion recovery; LGI-1: leucine-rich glioma-inactivated 1

Routine EEG findings were normal. Imaging studies, including color Doppler ultrasound of the urinary system, prostate, and male reproductive system, as well as CT scans of the thymus and lungs, revealed no mass lesions. A follow-up brain MRI, including both plain and contrast-enhanced scans, showed abnormal signal patterns in the bilateral hippocampi, with slightly low signal on T1-weighted sequences and hyperintensity on T2-weighted and fluid-attenuated inversion recovery (FLAIR) sequences. No significant enhancement was observed on the contrast-enhanced scan (Figures 2A-2D). Based on these findings, the diagnosis of anti-LGI-1 antibody-associated encephalitis was established according to three diagnostic pillars: (1) a compatible clinical syndrome comprising subacute cognitive impairment, FBDS, and psychiatric symptoms; (2) positive LGI-1 antibodies in both serum (titer of 1:320) and CSF (titer of 1:10) detected by CBA; and (3) supportive MRI findings showing bilateral hippocampal T2/FLAIR hyperintensity indicative of limbic inflammation.

Notably, the absence of abnormalities on the initial MRI and normal CSF findings did not exclude the diagnosis, highlighting an important clinical learning point from this case. The treatment regimen included methylprednisolone pulse therapy consisting of 1000 mg for three days, followed by 500 mg for three days, and then 240 mg for three days. This was subsequently followed by oral prednisone at 60 mg/day, with the dose reduced by 5 mg every two weeks. By the sixth day of pulse therapy, the patient's symptoms, including involuntary facial tics, right upper limb tics, impaired memory and calculation abilities, and sleep disturbances, began to improve. The patient was discharged on January 14, 2025. After discharge, he continued oral steroid therapy with a gradual tapering schedule while continuing antiepileptic treatment.

The steroids were discontinued six months later, and the antiepileptic medication was gradually tapered and ultimately discontinued nine months after discharge. This protocol was designed to rapidly eliminate pathogenic antibodies through high-dose pulse therapy, followed by a gradual oral taper to prevent rebound inflammation. Concurrent antiepileptic therapy with sodium valproate was maintained during the steroid taper and was gradually withdrawn based on the patient's clinical response. At the one-month follow-up after discharge, the patient's symptoms had almost completely resolved. By the 12-month follow-up, the patient had returned to work without limitations. At the most recent follow-up in April 2026, the patient's condition remained stable, with no signs of recurrence.

## Discussion

LGI-1 is a secretory synaptic protein that binds to ADAM23 on the presynaptic membrane and to ADAM22 on the postsynaptic membrane, thereby forming a stable "LGI-1-ADAM22/23" cross-synaptic complex. This complex maintains neuronal excitability homeostasis by bidirectionally regulating the presynaptic voltage-gated potassium channel (Kv1.1) and the postsynaptic α-amino-3-hydroxy-5-methyl-4-isoxazolepropionic acid receptor (AMPAR) [6,7]. Anti-LGI-1 antibodies primarily target N-terminal and C-terminal epitopes of the LGI-1 protein, disrupting the stability of the LGI-1-ADAM complex, leading to downregulation of postsynaptic Kv1.1 potassium channel density and a lowered depolarization threshold of the neuronal membrane potential, ultimately causing neuronal hyperexcitability [7].

The LGI-1 protein is highly expressed in the hippocampus, amygdala, and adjacent medial temporal structures [6]. The parahippocampal gyrus and uncus contain the primary olfactory cortex, which receives direct projections from the olfactory tracts and is responsible for odor recognition and emotional associations. In this case, the other typical clinical manifestations did not emerge until six months after the onset of olfactory hallucinations, suggesting that the antibodies initially selectively affected the uncus and adjacent limbic system. Paroxysmal olfactory hallucinations, particularly those involving unpleasant odors, are a classic manifestation of uncus seizures and are commonly observed in mesial temporal lobe epilepsy, uncus tumors, or limbic encephalitis [10].

However, reports of olfactory hallucinations as the initial or core symptom of anti-LGI-1 encephalitis are relatively rare. In our patient, the six-month interval between the onset of olfactory hallucinations and the emergence of cognitive impairment and FBDS demonstrated this prodromal pattern. A cohort study involving 45 patients at Beijing Tiantan Hospital showed that 24.4% of patients experienced focal episodes of lucidity, which may present with symptoms such as olfactory hallucinations and limb numbness [1]. Wang et al. [10] reported a case of anti-AMPAR2 encephalitis accompanied by olfactory hallucinations. These data indicate that olfactory hallucinations may occur in this condition; however, due to their lack of specificity, they are easily misinterpreted as auras associated with primary epilepsy or psychiatric symptoms, leading to diagnostic delays. In this case, the patient's olfactory hallucinations persisted for six months before a definitive diagnosis was made following the onset of FBDS and cognitive impairment.

In the spectrum of prodromal symptoms of LGI-1 encephalitis reported by Naasan et al. [4], anxiety, mood instability, and hallucinations may appear weeks to months before the development of full-blown encephalopathy. Therefore, olfactory hallucinations may represent one of the earliest prodromal symptoms of anti-LGI-1 encephalitis, appearing weeks to months before the onset of FBDS and cognitive impairment, and may serve as an important early warning sign. Consequently, in middle-aged and elderly patients, the recent onset of paroxysmal olfactory hallucinations, especially those involving unpleasant odors, should raise a high level of suspicion for anti-LGI-1 encephalitis, even when early brain MRI findings are negative and CSF results are normal. Antibody screening should therefore be performed as early as possible.

It is particularly noteworthy that 64% of patients with anti-LGI-1 encephalitis experience psychiatric symptoms, with personality changes (65.6%), hallucinations (46.9%), and mood disturbances (43.8%) being the most common manifestations. Among these patients, 56.25% presented with psychiatric symptoms as their initial manifestation, and 28.1% were first diagnosed in a psychiatric department [5]. These data indicate that hallucinations, mood disturbances, and personality changes are not merely incidental accompanying symptoms, but rather direct manifestations of functional disturbances resulting from early limbic system involvement.

In addition, brain MRI and anti-LGI-1 antibody testing play crucial roles in the diagnosis and differential diagnosis of this disease. According to previous reports, up to 46% of patients with anti-LGI-1 encephalitis have completely normal early MRI findings, with bilateral temporal lobe hyperintensity observed in only 27% of cases [11]. In this case, the initial MRI revealed no abnormalities, but follow-up imaging performed as the disease progressed showed bilateral hippocampal T2/FLAIR hyperintensity, demonstrating a dynamic evolution. This progressive imaging change reminds us that a negative early MRI should never rule out the diagnosis; in cases of high clinical suspicion, follow-up imaging should be actively pursued. Furthermore, unlike anti-NMDAR encephalitis, which commonly presents with CSF lymphocytosis and elevated protein levels, anti-LGI-1 encephalitis is characterized by normal CSF cell counts and biochemical parameters in over 70% of cases [12].

Consistent with previous literature, the patient's CSF pressure, cell count, protein, glucose, and chloride levels were all normal, and both microbiological and NGS tests were negative. Relying solely on CSF findings could easily lead to a misdiagnosis of a "non-organic" or "functional" disorder. Thus, normal CSF findings cannot exclude anti-LGI-1 encephalitis, and antibody testing remains the cornerstone of diagnosis. In our patient, the serum antibody titer of 1:320 was significantly higher than the CSF titer of 1:10, demonstrating a serum-predominant distribution, consistent with previous reports [3]. Additionally, hyponatremia serves as an important supportive clue. According to previous literature, the incidence of hyponatremia in patients with anti-LGI-1 encephalitis is approximately 50% to 60%. The mechanism involves the syndrome of inappropriate antidiuretic hormone secretion (SIADH), which is associated with involvement of the hypothalamic osmoregulatory center or the limbic system [13]. At the time of initial admission, the patient's serum sodium level was 135.4 mmol/L. Although this value was only slightly below the lower limit of normal, its presence alongside newly developed "epileptiform" symptoms in a middle-aged man should prompt a high degree of suspicion for autoimmune encephalitis.

Cognitive impairment in anti-LGI-1 encephalitis primarily affects recent memory and episodic memory, which is closely associated with structural and functional involvement of the hippocampus and medial temporal lobe [14]. In our patient, memory impairment and impaired calculation ability (unable to complete the task of subtracting 7 from 100 repeatedly) were evident. Following treatment, symptoms resolved completely, indicating that cognitive impairment in anti-LGI-1 encephalitis is reversible. This reversibility may be explained by the fact that antibody-mediated inflammation primarily causes synaptic dysfunction and reversible neuronal damage, rather than irreversible neuronal degeneration and death [15]. Therefore, early clearance of pathogenic antibodies and suppression of the inflammatory response can interrupt the cascade of damage, leading to complete recovery of cognitive function. Furthermore, existing literature [16] has indicated that in some patients, delayed treatment or persistent inflammation may result in hippocampal sclerosis and permanent memory impairment, further highlighting the urgency of early intervention.

Therefore, once anti-LGI-1 encephalitis is diagnosed or strongly suspected based on clinical findings, immunotherapy should be initiated immediately without waiting for tumor screening results. Unlike anti-NMDAR encephalitis, anti-LGI-1 encephalitis has a very low association with tumors (less than 5%, primarily thymomas); therefore, immunotherapy should not be delayed pending completion of tumor screening [17]. While corticosteroids remain the cornerstone of first-line treatment, it is important to note that steroid-sparing agents, such as rituximab and cyclophosphamide, may be considered in relapsing or refractory cases. Azathioprine or mycophenolate mofetil may also be used for maintenance therapy to prevent recurrence. Additionally, intravenous immunoglobulin (IVIG) or plasma exchange may serve as adjunctive or alternative first-line options in selected patients [8,18].

The multicenter cohort study conducted by Seery et al. provides strong evidence supporting various treatment strategies. The study showed that methylprednisolone pulse therapy is independently associated with improvement in the 12-month Modified Rankin Scale (mRS) score and favorable outcomes, and that initiating treatment within three months is significantly associated with mRS improvement [8]. Notably, Thompson et al. [18] found that FBDS is highly time-sensitive to immunotherapy: 51% of patients experienced resolution of FBDS within 30 days of initiating first-line immunotherapy, whereas antiepileptic drugs alone were effective in only 10% of cases. Additionally, the recurrence rate of anti-LGI-1 encephalitis is 30%, with a median time to recurrence of 414 days (approximately 14 months), suggesting that recurrence can occur long after treatment [18]. According to Yi et al. [5], the 12-month recurrence rate was as high as 53.1% in patients with psychiatric symptoms, significantly higher than the 16.7% rate in those without such symptoms. These patients also exhibited poorer functional independence. Therefore, patients with LGI-1 encephalitis should undergo at least two to three years of follow-up to allow early detection of potential recurrences or sequelae.

We acknowledge that drawing broad conclusions from a single case report has inherent limitations. However, case reports play a crucial and valuable role in the medical literature, particularly in documenting rare or novel presentations, such as olfactory hallucinations as an early symptom. Such presentations may be overlooked in larger cohort studies. The significance of this case lies in its emphasis on olfactory hallucinations as a potentially underrecognized early warning sign of anti-LGI-1 encephalitis. Recognizing this symptom may facilitate earlier antibody screening and diagnosis in future patients.

## Conclusions

Olfactory hallucinations may represent an early warning sign or potential prodromal manifestation of anti-LGI-1 antibody-associated encephalitis, often appearing before the onset of FBDS and cognitive impairment. In middle-aged and elderly patients, the presence of new episodic unpleasant olfactory hallucinations, along with hyponatremia, mild anxiety or depression, or abnormal EEG findings, should raise suspicion of this condition, even in the presence of a negative brain MRI or normal CSF findings. Antibody screening via CBA is crucial for diagnosis, as normal early MRI and CSF findings do not exclude the disorder. This autoimmune condition results from antibodies targeting the LGI-1 protein, leading to neuronal hyperexcitability. Current evidence suggests that early glucocorticoid pulse therapy, ideally initiated within three months of symptom onset, particularly after the development of FBDS, is associated with rapid clinical improvement and better functional outcomes. If treatment is initiated before irreversible hippocampal damage occurs, complete cognitive recovery is possible.
